# Sex differences in human deep grey matter microstructure emerge during adolescence

**DOI:** 10.1016/j.dcn.2026.101811

**Published:** 2026-09-05

**Authors:** Christopher G. Filippi, Jay N. Giedd, Aziz M. Uluğ, Richard Watts

**Affiliations:** a The Hospital for Sick Children, Canada; b The SickKids Research Institute, Canada; c University of Toronto, Canada; d Department of Psychiatry, University of California San Diego, La Jolla, CA, USA; e Institute of Biomedical Engineering, Boğaziçi University, Istanbul, Turkey; f Cortechs Labs, Inc., San Diego, USA; g Faculty of Health, University of Canterbury, Christchurch, New Zealand

**Keywords:** Brain, Sex differences, Development, Diffusion MRI, Puberty

## Abstract

Many neuropsychiatric disorders demonstrate sex-based differences in their prevalence, onset age, and phenotypic presentation. The neuroanatomic and neurophysiologic substrates that underpin these sex differences are not fully understood. Animal studies have demonstrated sex differences in neuronal, dendritic, and glial microstructure. Restriction Spectrum Imaging (RSI) is an advanced diffusion MR technique that delineates the intracellular, extracellular, and free water contributions to diffusion signal, providing unique insights into microstructure. A recent RSI study showed large sex differences in subcortical gray matter structures in young adults, most significantly in the hippocampus, amygdala, and nucleus accumbens, which persist when controlling for age and volume. In this study, we use RSI to examine sex-related cross-sectional developmental patterns in subcortical gray matter across childhood and adolescence into early adulthood by combining the Human Connectome Project–Development and Young Adult datasets (ages 9–35 years). We observe that sex differences emerge over adolescence, with males showing higher restricted diffusion and lower hindered diffusion than females across all deep gray structures interrogated. We establish sex-stratified MR diffusion based normative age curves, for subcortical gray matter that will support future neurodevelopmental research.

## Highlights

• Microstructure in hormone-sensitive limbic–striatal nuclei diverge between males and females during adolescence.
• We present cross-sectional normative development curves for males and females.
• Sex differences in brain development may shed light on differences in the onset and prevalence of mental health disorders.

## Graphical abstract

**Figure:**
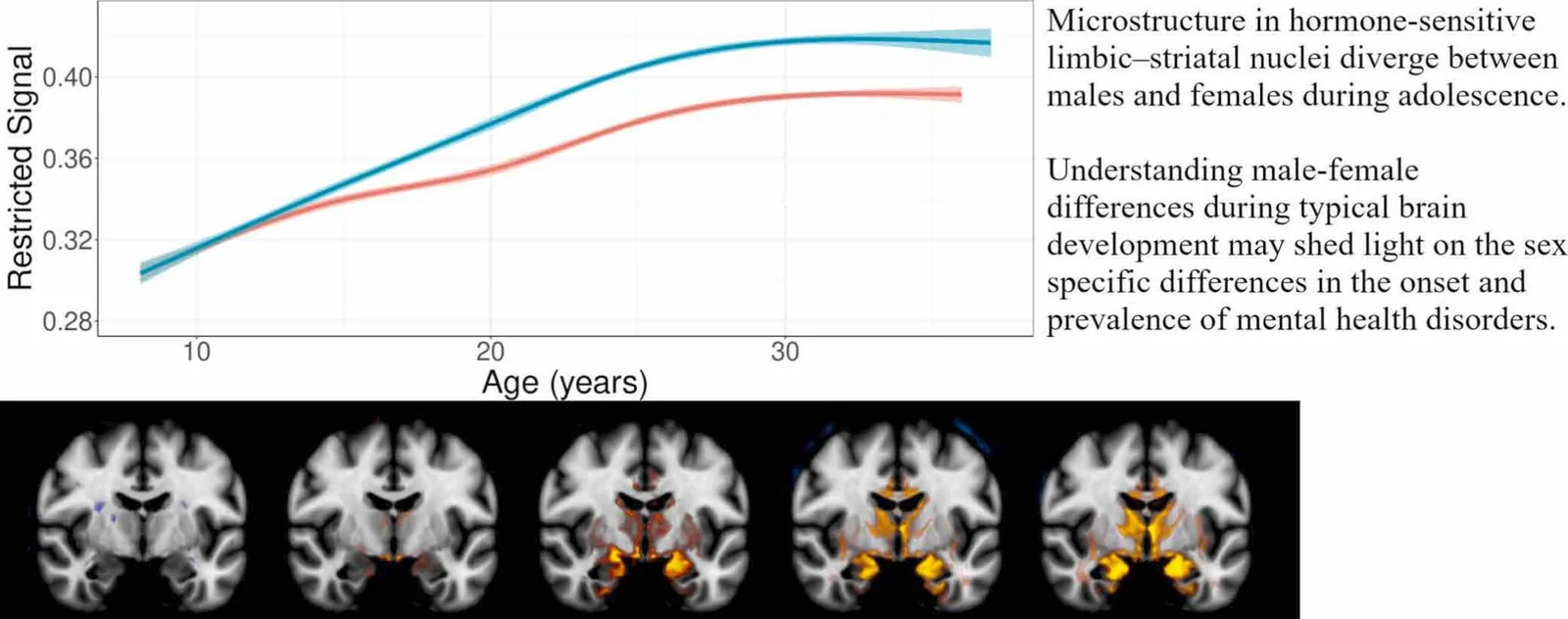

## 1 Introduction

There are sex-differences in the incidence and prevalence of many neuropsychiatric disorders, with autism and attention problems being more common in males (Loomes et al., 2017, Polanczyk et al., 2007) while depression and anxiety are more common in females (McLean et al., 2011, Salk et al., 2017). It remains unclear whether this is driven by neurophysiologic and/or neuroanatomic changes as the brain develops. Animal models have shown that sex-differences exist in the microstructure of subcortical gray matter including changes in neuronal, dendritic, and glial microstructure. Human studies have primarily used magnetic resonance imaging (MRI) to study sex-related differences in brain morphometry.

Large-scale cohort studies have shown sex-related differences, specifically larger brain volumes in adolescent males, including subcortical gray matter, even when adjusted for total brain size (Williams et al., 2021a). Another large-scale study of harmonized data from 100 MR studies and over 120,000 scans showed that subcortical gray matter volume peaks in adolescence at 14.4 years of age with a subsequent decline in both sexes with between subject variability peaking in late adolescence (Bethlehem et al., 2022). In a study using data from the UK Biobank with over 40,000 participants, two-thirds of brain measures showed significant sex differences, even after controlling for global brain size, although effect sizes were small (Williams et al., 2021b). In the same study, sex differences in global brain size could not be solely explained by weight or height.

A mega-analysis study (Wierenga et al., 2022) investigated sex differences in brain structure with over 16,600 individuals across the lifespan and found that there is greater male variance across all subcortical measures (volume, cortical thickness, and cortical surface area), 50% of subcortical structures retain these sex differences across the lifespan, and the variance in subcortical volumes was greater in males. In a study using ABCD data from ages 9–11, sex was a predictor of early adolescent subcortical volume and microstructure, and there was greater male variability in the volumes of the hippocampus, pallidum, and putamen (Torgerson et al., 2025). While other studies using ABCD have shown individual differences that vary by age, pubertal status, and brain regions (Herting et al., 2018, Tamnes et al., 2017), others have shown more limited support for greater male variability (Bottenhorn et al., 2023).

Less is known about the microstructural features underlying these volumetric differences. Restriction spectrum imaging (RSI) is a multicompartment model of the diffusion MRI signal that has been validated in animal models (White et al., 2013). The measured signal is modeled as a sum of the signals from restricted, hindered, and free water. Intracellular water, with low diffusivity, contributes to the restricted compartment, while interstitial water is modeled by the hindered compartment, yielding histologically anchored markers of cellular density and the extracellular milieu (White et al., 2013). Recent work has shown there are large sex differences in RSI metrics in subcortical gray matter in young adults, most significantly in the hippocampus, amygdala, and nucleus accumbens, which persisted when controlling for age and volume (Pecheva et al., 2024).

## 2 Materials and Methods

### 2.1 Data Source

HCP-YA data were taken from the 1200 Subjects Data Release (March 1, 2017), while HCP-D data were from the Lifespan 2.0 release (Feb 26, 2021). Adults provided written informed consent, while for children written consent was obtained from their guardian and assent from the participant.

### 2.2 Image Acquisition Protocols

The imaging protocols for the HCP-YA and HCP-D studies have been described previously (Harms et al., 2018, Sotiropoulos et al., 2013, Van Essen et al., 2013). Both protocols use a spin-echo EPI acquisition with matched TE= 89ms. HCP-YA data were acquired using a customized Siemens 3 T Connectom scanner with 100 mT/m gradients, TR = 5520 ms, multiband factor of 3, isotropic 1.25 mm resolution, 90 non-colinear diffusion weighting directions for each of three shells (b=1000, 2000, 3000 s/mm^2^) and 18 acquisitions with b= 0. This was repeated with L/R and R/L phase-encoding directions to allow correction of susceptibility-induced distortions (Sotiropoulos et al., 2013, Van Essen et al., 2013).

The protocol for HCP-D was shortened to be suitable for use in a pediatric population while maintaining adequate signal-to-noise ratio (Harms et al., 2018). Data were acquired from four sites, each using a Siemens 3 T Prisma scanner with 80 mT/m gradients, TR = 3230 ms, multiband factor of 4, isotropic 1.5 mm resolution, 92–93 non-colinear directions for each of two shells (b=1500, 3000 s/mm^2^), and 14 acquisitions with b= 0. This was repeated with A/P and P/A phase-encoding directions (Harms et al., 2018). Only data passing HCP recommended quality control standards based on image SNR, participant motion, and site consistency were included in the released HCP-YA and HCP-D data. No further QC was applied in this study.

### 2.3 Image Processing

HCP-YA diffusion data were taken from the Diffusion Preprocessed package. For HCP-D, preprocessed diffusion data were not available, so data from the Diffusion Unprocessed package was used and preprocessed using the HCP preprocessing pipeline version 4.3.0 (Glasser et al., 2013). Diffusion preprocessing in both cases included correction for susceptibility and eddy-current induced EPI distortions and participant motion using FSL eddy, gradient non-linearity correction, and rigid body registration to the structural T1- and T2-weighted images.

The RSI model was fitted using the previously published parameters (Hagler et al., 2019) using RSIproc, available at https://github.com/ABCD-STUDY/RSI. The restricted and hindered compartments were modeled as both having longitudinal diffusivities of 1.0 × 10^−3^ mm^2^/s and transverse diffusivities of 0.0 and 0.9 × 10^−3^ mm^2^/s, respectively, with the free fluid component having an isotropic diffusivity of 3 × 10^−3^ mm^2^/s. Diffusion metrics were normalized by the L2-norm of the spherical harmonics for all components (Hagler et al., 2019).

The RSI model provides measures of the signal fractions attributed to the isotropic and directional components of the restricted and free compartments, along with the isotropic free water compartment. Since the grey matter regions investigated are largely isotropic due to directional averaging of neurite orientations, we chose to concentrate our analyses on the restricted and hindered isotropic components, corresponding to low and intermediate diffusivities. In the absence of substantial free water and directional components, the restricted and free components are expected to be inversely correlated due to the signal normalization.

For region-of-interest analysis, Freesurfer-derived subcortical grey matter segmentations were used to define subcortical structures, with the RSI metrics resampled into the same native T1-weighted imaging space. Homologous left and right hemisphere ROIs were averaged prior to analysis.

For voxelwise analysis, RSI data were nonlinearly transformed into the space of the Montreal Neurological Institute (MNI) template using the FSL. Transformed maps were then smoothed with a 1.0 mm (sigma) Gaussian kernel.

### 2.4 Modelling

ROI-based analyses were performed in R using linear mixed effects models to account for relatedness. To account for offsets in the metrics derived from the two protocols, the following linear model was applied to the data from both protocols.

RSImetric ∼ ns(age) + sex + ns(age)*sex + protocol

Where ns represents a basis matrix for natural cubic splines with knots at ages 15, 20, and 25. Supplemental Figures S1-S5 show the raw RSI metrics for the two protocols before and after correction for the protocol offsets. Sex differences within discrete 2-year age ranges were calculated using a linear model with age, sex, and BMI as covariates. For HCP-YA data (22–35 years), a mixed-effects model with family ID as a random effect was used to account for relatedness in this sample that is enriched for twins.

Voxelwise analysis of imaging data was performed using the Fast and Efficient Mixed Effects Analysis (FEMA) package (Parekh et al., 2024). Data were stratified into five age ranges: 9–12, 13–16, 17–21, 22–28, and 29–36 years, with the first three ranges covering HCP-D, and the final two HCP-YA. Over these restricted ranges, the variation of the signal was modeled as a linear function of age. Standardized effect sizes were estimated by dividing the effect size of sex (beta) by the standard deviation of the residuals from the model for each age range. Beta values, z-statistics, and standardized effect size maps are available on the Brain Analysis Library of Spatial maps and Atlases (BALSA) database (balsa.wustl.edu).

The proportion of variance uniquely explained by sex differences for each predefined ROI was quantified for each of these age ranges as the partial R^2^ for sex. This represents the fractional reduction in the residual sum of squares when sex was added to a model containing age and BMI (Table 2). The statistical significance of the contribution of sex to the model was assessed using an F-test. P-values were Bonferroni-corrected for the number of tests performed within each analysis (n_Tests_ = 60 for Table 2).

To determine whether puberty predicts RSI metrics independently of age within the HCP-D cohort, ROI models were run with and without the Tanner composite score, and the individual subscores (height spurt, body hair, skin/acne for both males and females, voice deepening and facial hair for males only, breast development and menarche for females only).

## 3 Results

Demographics for each age group are shown in Table 1.

**Table 1:** Participant demographics by age range and study.

**Age Range**	**9–12**	**13–16**	**17–21**	**22–28**	**29–36**
**Total N (%)**	181 (11)	221 (13.5)	177 (10.8)	506 (30.8)	558 (34.0)
**Age Mean (SD)**	10.8 (1.2)	14.8 (1.2)	19.7 (1.4)	25.5 (1.9)	31.7 (2.0)
**Sex Male (%)**	82 (45.3)	107 (48.4)	82 (46.3)	283 (55.9)	206 (36.9)
**BMI Mean (SD)**	18.2 (3.4)	21.8 (4.4)	24.0 (4.9)	26.1 (4.8)	26.6 (5.4)
**Study/Protocol**	HCP-D	HCP-D	HCP-D	HCP-YA	HCP-YA

**Fig. 1:**
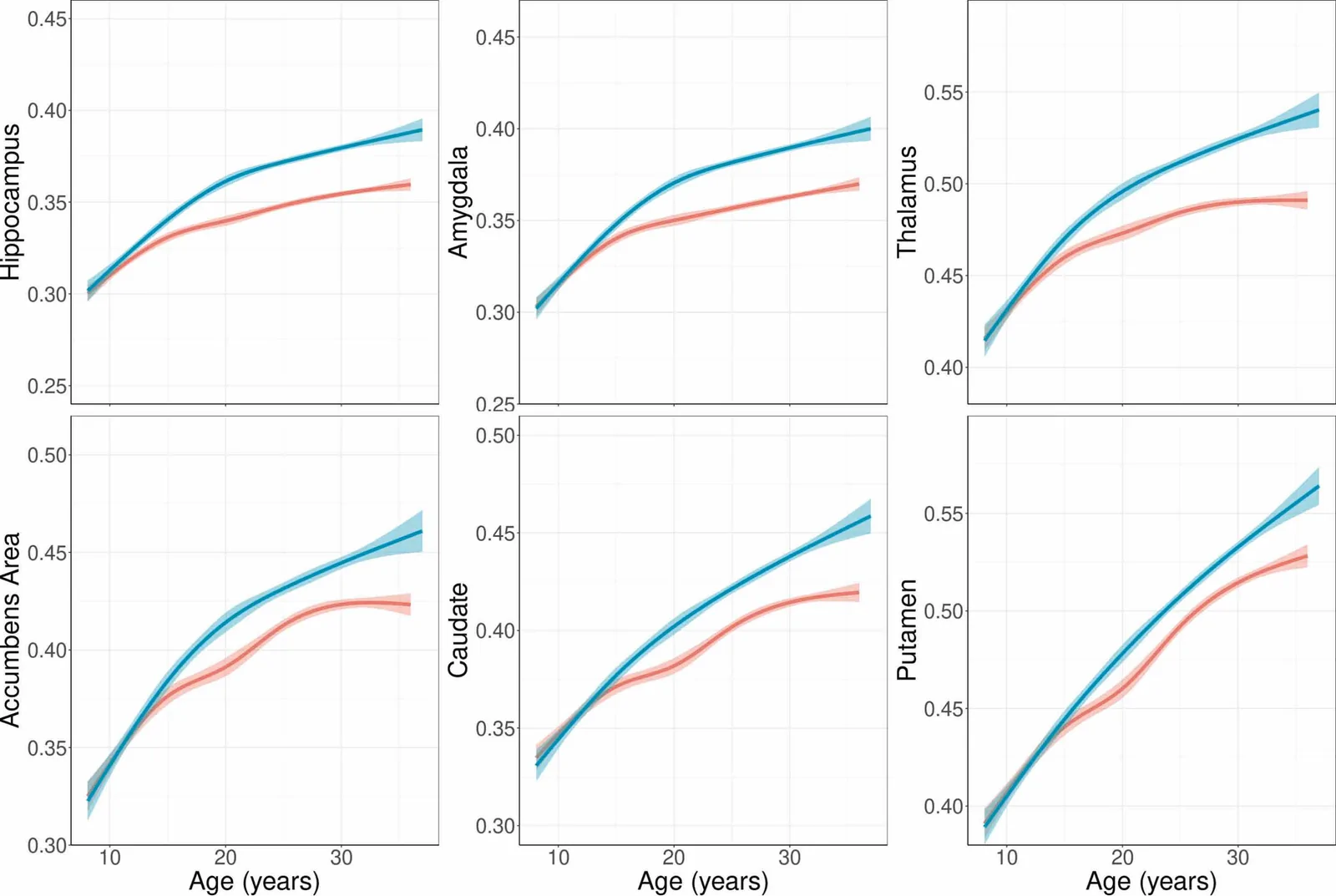
Variation of restricted normalized isotropic (RNI) component of diffusion with age and sex (red=female, blue=male) after protocol-offset correction in each of the Freesurfer-derived ROIs.

The restricted component of diffusion increased monotonically with age (Fig. 1). In the nucleus accumbens and thalamus, the restricted signal reached a plateau in the females around age 30–35, while the values for males continued to increase. In the hippocampus and amygdala, the restricted component was still increasing for both sexes at age 35, although the hindered component largely stabilized in the early 20 s (supplemental Figure S5).

**Fig. 2:**
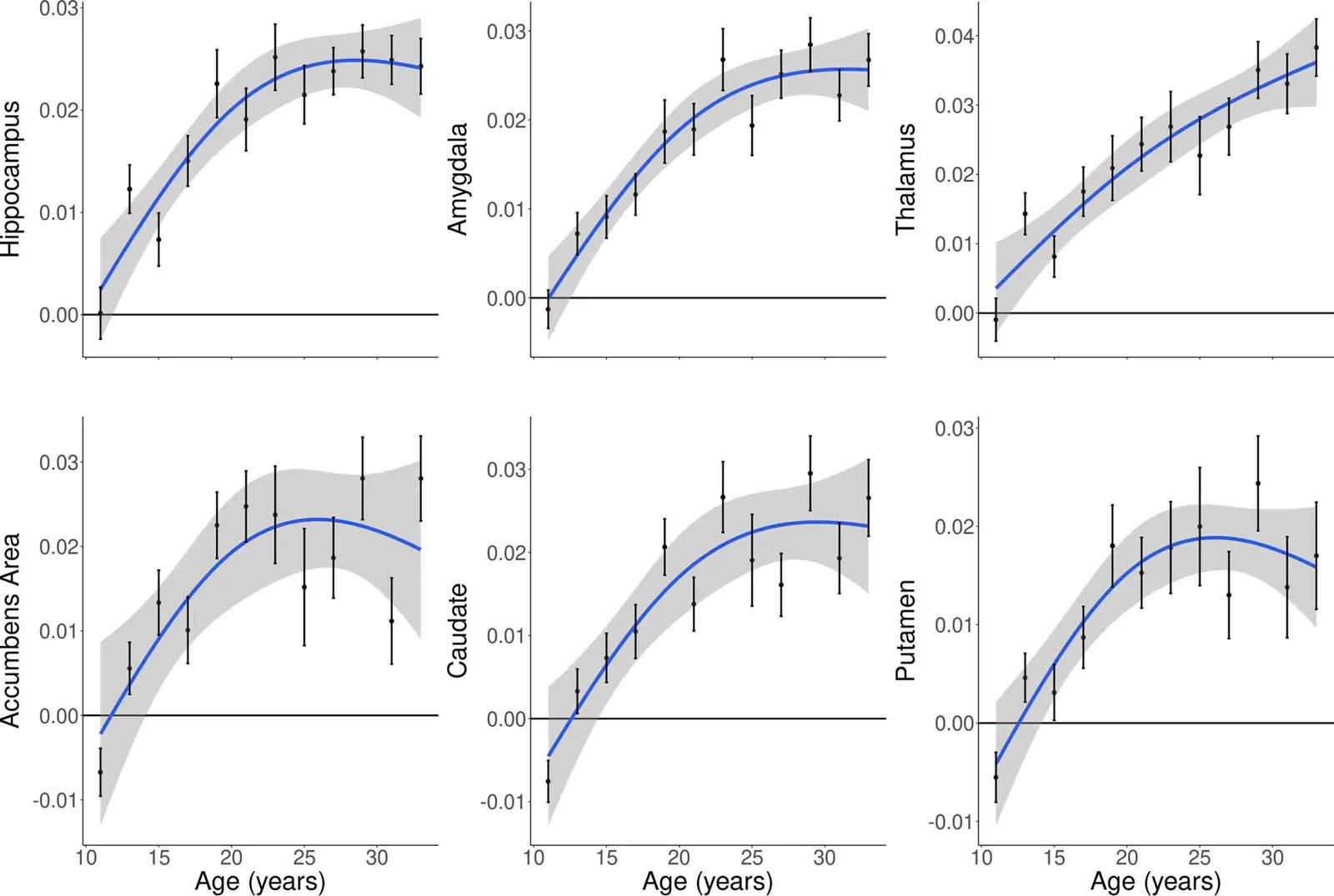
Sex differences (males−females) in restricted component of diffusion across discrete 2-year age bins in each of the Freesurfer-derived ROIs.

**Fig. 3:**
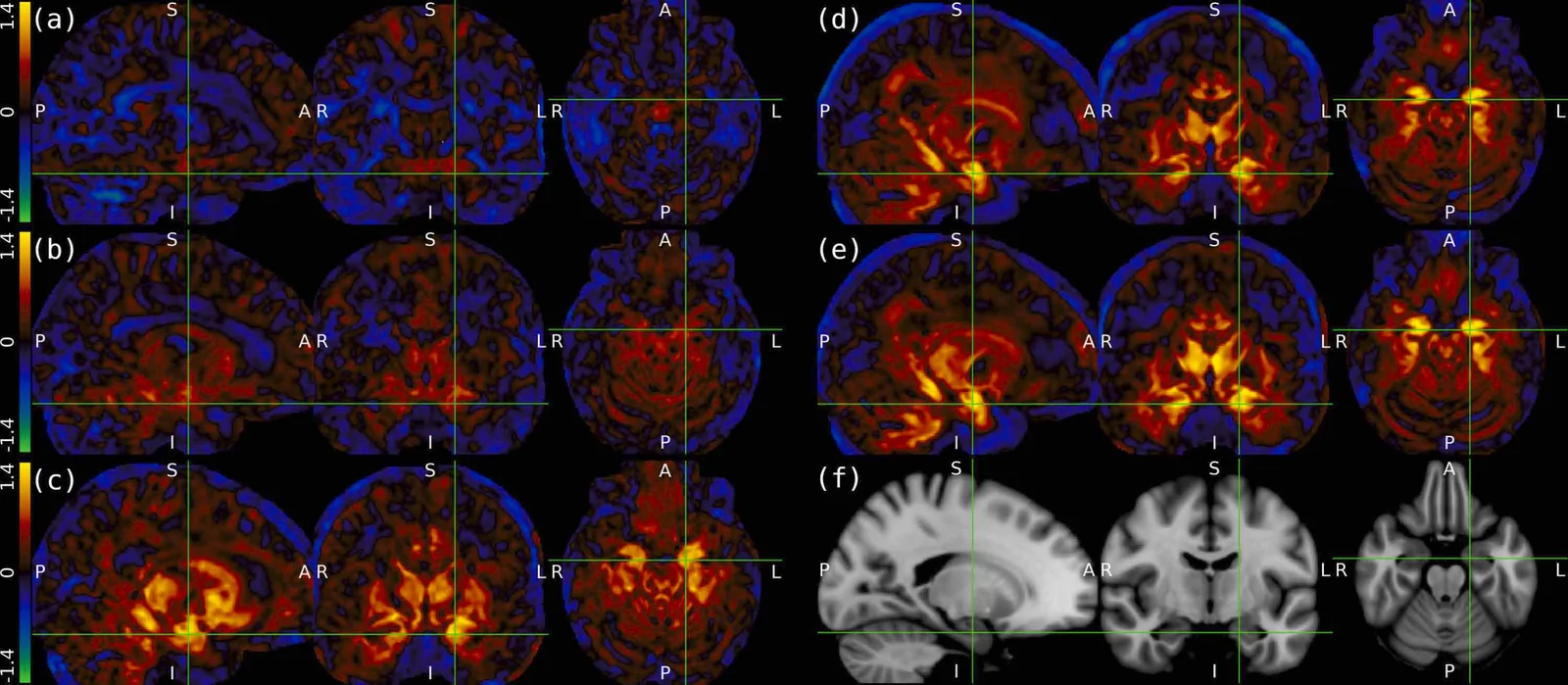
Voxelwise analysis of sex differences in the restricted normalized isotropic (RNI) component of diffusion for age ranges (a) 9–12, (b) 13–16, (c) 17–21, (d) 22–28, and (e) 29–36 years. Red-yellow indicates greater RNI in males, while blue-green indicates greater RNI in females. Scale bar indicates standardized effect size. (f) Corresponding T1-weighted image for reference.

At the youngest age, sex differences were minimal (Fig. 2, Fig. 3, statistics for ROI-based analyses provided in Supplementary Table S1) but developed over puberty. Consistent across all regions, from puberty, males showed greater restricted component of diffusion than females, while females showed a greater hindered component than males. In the nucleus accumbens, caudate, and putamen, females showed a slowing in development in their late teens, before accelerating again in their early 20 s. Males showed more monotonic development in all regions. The sex differences in the amygdala and hippocampus reached a steady state around age 25, while other regions showed continuing separation. The thalamus showed almost a linear divergence with age, while the remaining regions showed indications of approaching a plateau by age 35.

Table 2 shows that sex differences explain an increasing proportion of variance in RNI with age. At ages 9–12, none of the regions investigated showed significant sex differences after correction for multiple comparisons (p_Bonferroni_ > 0.05). Between ages 13–16, sex explained significant variance (p_Bonferroni_ < 0.001) in the hippocampus, amygdala and thalamus, while by age 17–21, sex accounted for 13–33% of RNI variance across all regions (p_Bonferroni_ < 0.001). These effects persisted into adulthood, especially in the hippocampus and amygdala (partial R^2^ of 32–37%). HNI showed a complementary relationship, explaining similar proportions of variance.

**Table 2:** Partial R² of sex for RNI and HNI by region and age group. Values show the proportion of variance uniquely explained by sex after controlling for age and BMI, with Bonferroni-corrected F-test p-values in parentheses (60 comparisons: 2 metrics × 6 regions × 5 age groups).

**Restricted Normalized Isotropic (RNI)**					
**Region**	**9–12**	**13–16**	**17–21**	**22–28**	**29–36**
Accumbens	0.000 (1.000)	0.073 (0.003)	0.273 (7.1 ×10^−12^)	0.084 (2.2 ×10^−9^)	0.130 (2.3 ×10^−16^)
Amygdala	0.001 (1.000)	0.170 (1.4 ×10^−8^)	0.348 (5.1 ×10^−16^)	0.317 (2.5 ×10^−41^)	0.341 (3.3 ×10^−49^)
Caudate	0.003 (1.000)	0.039 (0.193)	0.125 (9.9 ×10^−5^)	0.152 (8.4 ×10^−18^)	0.168 (1.1 ×10^−21^)
Hippocampus	0.046 (0.231)	0.175 (6.1 ×10^−9^)	0.329 (6.2 ×10^−15^)	0.371 (2.3 ×10^−50^)	0.364 (2.1 ×10^−53^)
Putamen	0.011 (1.000)	0.030 (0.621)	0.193 (6.8 ×10^−8^)	0.081 (5.3 ×10^−9^)	0.084 (3.6 ×10^−10^)
Thalamus	0.011 (1.000)	0.131 (2.3 ×10^−6^)	0.277 (4.3 ×10^−12^)	0.206 (4.6 ×10^−25^)	0.328 (8.6 ×10^−47^)
**Hindered Normalized Isotropic (HNI)**					
**Region**	**9–12**	**13–16**	**17–21**	**22–28**	**29–36**
Accumbens	0.009 (1.000)	0.042 (0.146)	0.163 (1.8 ×10^−6^)	0.150 (1.5 ×10^−17^)	0.232 (4.4 ×10^−31^)
Amygdala	0.022 (1.000)	0.169 (1.5 ×10^−8^)	0.311 (6.2 ×10^−14^)	0.156 (2.5 ×10^−18^)	0.237 (7.0 ×10^−32^)
Caudate	0.022 (1.000)	0.026 (0.950)	0.053 (0.126)	0.243 (3.7 ×10^−30^)	0.268 (7.7 ×10^−37^)
Hippocampus	0.000 (1.000)	0.073 (0.003)	0.257 (4.9 ×10^−11^)	0.320 (8.0 ×10^−42^)	0.351 (5.1 ×10^−51^)
Putamen	0.002 (1.000)	0.046 (0.083)	0.177 (3.8 ×10^−7^)	0.107 (3.4 ×10^−12^)	0.086 (1.7 ×10^−10^)
Thalamus	0.038 (0.509)	0.118 (1.1 ×10^−5^)	0.297 (3.9 ×10^−13^)	0.292 (2.0 ×10^−37^)	0.374 (2.8 ×10^−55^)

Supplemental Figures S6 and S7 show how the Tanner composite score and its subscores vary with age. Within the HCP-D cohort, the Tanner composite score was highly correlated with age, Pearson r = 0.818/0.842 for males/females (Supplementary Table S2). After Bonferroni correction, only the relationship between HNI in the thalamus and menarche in females showed significant association in a model including age and BMI (p_Uncorrected_ = 1.7 ×10^−4^, p_Bonferroni_ = 0.015). See also Supplementary Tables S3-S5.

## 4 Discussion

Our results show that sex differences in diffusion metrics are minimal before age 10 (Fig. 1, Table 2), but gradually emerge over adolescence, reaching something of a steady state (particularly in the hippocampus and amygdala) before age 20. This developmental period coincides with puberty, suggesting a potential role of sex hormones.

In the hippocampus, increased restricted signal in males aligns with animal work showing increased cell proliferation in CA1/CA3 and dentate gyrus (Bowers et al., 2010) and larger soma size (Isgor and Sengelaub, 2003). These differences are influenced by gonadal hormones localized to dendrites, synapses, and axon terminals (Loy et al., 1988, Shughrue et al., 1997, Simerly et al., 1990) and to glial processes (McEwen et al., 2012). In humans, the hippocampus is rich in estrogen alpha and estrogen beta receptors and progesterone receptors and central to memory; estradiol enhances synaptic plasticity and spine density (Woolley and McEwen, 1994).

Pubertal steroids modulate cell addition in the medial amygdala (Ahmed et al., 2008), with more synapses and receptor expression differences (Nishizuka and Arai, 1981, Österlund and Hurd, 2001, Page et al., 2022) that may account for the greater restricted signal in males, as absolute amygdala size is typically larger in males (DeCasien, A.R., Guma, E., Liu, S. & Raznahan, A., 2022, Ruigrok et al., 2014). After puberty, males show greater restricted diffusion in striatal nuclei, consistent with hormonally influenced maturation (Padmanabhan and Luna, 2014, Wahlstrom et al., 2010), and sex differences in dopaminergic/opioid receptor distribution in the nucleus accumbens (Sharp et al., 2022).

In our study, diffusion metrics diverge between males and females over adolescence, aligning with the emergence of sex-biased mood disorders (Amiel Castro et al., 2021). For example, adolescent increases in anxiety/depression are more pronounced in females (Altemus et al., 2014, Mir and Rivarola, 2022). In animal models, female rodents show greater immobility after puberty (Martinez-Mota et al., 2011) and physiologic estrogen drops increase anxiety and reduce dendritic density (Gouveia et al., 2004, van Veen, 2020). The ventral hippocampus underlies rodent anxiety-like behaviors (Bannerman et al., 2014, Felix-Ortiz et al., 2013, Jimenez et al., 2018, McHugh et al., 2004, Parfitt et al., 2017) and is modulated by pubertal hormones (Boivin et al., 2017, Goel and Bale, 2010). Changes in ventral hippocampus–nucleus accumbens circuitry contribute to sex-specific stress susceptibility (Muir et al., 2020, Williams et al., 2020). Likewise, dopamine systems show adolescent increases; dopamine receptor D1 peaks and D2 declines bias toward approach and risk (Andersen, 2003, Selemon, 2013). Globus pallidus volume typically decreases through adolescence with sex-specific trajectories (Goddings et al., 2014). Nucleus accumbens changes (dopamine bursts, μ-opioid receptor density) heighten motivational ‘wanting’ and hedonic ‘liking’ (Dahl, 2004), and estrogen enhances dopamine release contributing to sex-specific addiction vulnerability (Becker and Hu, 2008).

### 4.1 Limitations

Our study was designed to quantify diffusion metrics with RSI from childhood to early adulthood. Although there are clear sex-differences that emerge during adolescence and coincide with puberty, any potential role that sex steroids may play in the diffusion changes that we observed is speculative. We show that Tanner composite score highly correlates with age, limiting our statistical power to determine age-independent pubertal effects. After correction for multiple comparisons, only the hindered diffusion in the thalamus and menarche in females show a significant association, tying a discrete hormonal milestone to a specific microstructural change.

Protocol differences between HCP-YA and HCP-D were addressed via harmonization with protocol-dependent offsets (see Figures S1–S5). Our study used data from two cross-sectional studies to quantify normative age curves for males and females. Future longitudinal studies would enable the exploration of individual developmental trajectories and their relationship to mental health.

## 5 Conclusion

Our study demonstrated the emergence of sex differences in the microstructure of deep gray nuclei during adolescence. There was greater restricted isotropic (intracellular) diffusion in males whereas hindered diffusion signal was relatively higher in females. The timing and location of these diffusion changes is consistent with, but not conclusive of the effects of pubertal endocrine activation and hormone receptor-density profiles. These different normative age curves may provide imaging markers of sex-biased vulnerability for mental health disorders that may ultimately inform developmentally targeted prevention and treatment.

## CRediT authorship contribution statement

**Aziz M. Uluğ:** Writing – review & editing, Validation, Software, Methodology, Formal analysis, Conceptualization. **Richard Watts:** Writing – review & editing, Writing – original draft, Software, Methodology, Investigation, Formal analysis, Data curation, Conceptualization. **Christopher G. Filippi:** Writing – review & editing, Writing – original draft, Resources, Project administration, Investigation, Conceptualization. **Jay N. Giedd:** Writing – review & editing, Writing – original draft, Validation, Formal analysis.

## Declaration of Competing Interest

The authors declare that they have no known competing financial interests or personal relationships that could have appeared to influence the work reported in this paper.

## Data Availability

The data that support the findings of this study are available from WU-Minn HCP Consortium. Restrictions apply to the availability of these data, which were used under license for this study. NDA/NIMHHCP Development
BALSAHCP Young Adult
