# Supplementary material for: Sex differences in human deep grey matter microstructure emerge during adolescence
Source: Dev Cogn Neurosci. 2026 Sep 5;81:101811. doi: 10.1016/j.dcn.2026.101811 (PMC13583605; doi:10.1016/j.dcn.2026.101811)
Supplement: Supplementary file 1 [file mmc1.docx]

**Supplementary Material**

**Supplementary Table S1.** Sex differences (male − female) in RNI and HNI across 2-year age bins. Values show β (SE). Significance markers reflect Bonferroni correction for 144 comparisons (2 metrics × 6 regions × 12 age bins): * p_Bonferroni_ < 0.05, ** p_Bonferroni_ < 0.01, *** p_Bonferroni_ < 0.001. Green shading = p_Bonferroni_ < 0.001. Age bins 10–22 years: HCP-D (linear regression); 22–34 years: HCP-YA (linear mixed-effects with family random effect). Positive β = males > females; negative β = females > males.

| **Restricted Normalized Isotropic (RNI) — Males minus Females** | | | | | | |
| --- | --- | --- | --- | --- | --- | --- |
| **Age range** | **Accumbens** | **Amygdala** | **Caudate** | **Hippocampus** | **Putamen** | **Thalamus** |
| 10–12 | -0.006 (0.003) | -0.002 (0.002) | -0.008 (0.003) | 0.001 (0.003) | -0.006 (0.003) | -0.001 (0.003) |
| 12–14 | 0.006 (0.003) | 0.007 (0.002) | 0.003 (0.004) | **0.013 (0.002)***** | 0.004 (0.002) | **0.014 (0.003)***** |
| 14–16 | 0.013 (0.004) | **0.009 (0.002)*** | 0.007 (0.004) | 0.007 (0.003) | 0.002 (0.003) | 0.008 (0.003) |
| 16–18 | 0.010 (0.004) | **0.012 (0.002)***** | 0.013 (0.004) | **0.015 (0.003)***** | 0.008 (0.003) | **0.017 (0.004)***** |
| 18–20 | **0.021 (0.005)**** | **0.018 (0.003)***** | 0.016 (0.005) | **0.021 (0.004)***** | **0.017 (0.004)*** | **0.019 (0.005)*** |
| 20–22 | **0.024 (0.004)***** | **0.019 (0.003)***** | 0.013 (0.004) | **0.019 (0.003)***** | **0.014 (0.003)*** | **0.022 (0.004)***** |
| 22–24 | **0.021 (0.006)*** | **0.027 (0.003)***** | **0.024 (0.004)***** | **0.025 (0.003)***** | 0.015 (0.004) | **0.025 (0.005)***** |
| 24–26 | 0.015 (0.007) | **0.019 (0.003)***** | 0.019 (0.005) | **0.021 (0.003)***** | 0.020 (0.006) | **0.023 (0.006)**** |
| 26–28 | **0.019 (0.005)*** | **0.025 (0.003)***** | **0.016 (0.004)**** | **0.024 (0.002)***** | 0.013 (0.004) | **0.027 (0.004)***** |
| 28–30 | **0.028 (0.005)***** | **0.029 (0.003)***** | **0.029 (0.004)***** | **0.026 (0.003)***** | **0.024 (0.005)***** | **0.035 (0.004)***** |
| 30–32 | 0.011 (0.005) | **0.023 (0.003)***** | **0.019 (0.004)***** | **0.025 (0.002)***** | 0.014 (0.005) | **0.033 (0.004)***** |
| 32–34 | **0.028 (0.005)***** | **0.027 (0.003)***** | **0.027 (0.005)***** | **0.024 (0.003)***** | 0.017 (0.005) | **0.038 (0.004)***** |
| **Hindered Normalized Isotropic (HNI) — Males minus Females** | | | | | | |
| **Age range** | **Accumbens** | **Amygdala** | **Caudate** | **Hippocampus** | **Putamen** | **Thalamus** |
| 10–12 | 0.001 (0.003) | -0.004 (0.003) | -0.009 (0.004) | 0.000 (0.004) | 0.003 (0.003) | -0.004 (0.004) |
| 12–14 | -0.007 (0.004) | -0.010 (0.004) | -0.013 (0.005) | -0.011 (0.004) | -0.004 (0.003) | **-0.020 (0.004)***** |
| 14–16 | -0.010 (0.004) | -0.009 (0.003) | -0.005 (0.006) | -0.010 (0.004) | -0.004 (0.003) | -0.009 (0.004) |
| 16–18 | 0.001 (0.004) | **-0.016 (0.003)***** | -0.005 (0.006) | **-0.016 (0.004)*** | -0.006 (0.004) | **-0.021 (0.005)*** |
| 18–20 | **-0.021 (0.005)*** | **-0.025 (0.005)***** | -0.024 (0.010) | **-0.029 (0.006)***** | **-0.023 (0.005)***** | **-0.033 (0.006)***** |
| 20–22 | **-0.021 (0.004)***** | **-0.025 (0.003)***** | -0.020 (0.008) | **-0.026 (0.005)***** | -0.014 (0.004) | **-0.034 (0.005)***** |
| 22–24 | **-0.029 (0.005)***** | **-0.021 (0.004)***** | **-0.032 (0.006)***** | **-0.032 (0.004)***** | **-0.024 (0.005)***** | **-0.052 (0.006)***** |
| 24–26 | -0.029 (0.009) | -0.014 (0.005) | **-0.040 (0.007)***** | **-0.028 (0.005)***** | **-0.029 (0.008)*** | **-0.042 (0.007)***** |
| 26–28 | **-0.022 (0.005)***** | **-0.022 (0.004)***** | **-0.031 (0.005)***** | **-0.031 (0.004)***** | -0.016 (0.005) | **-0.044 (0.005)***** |
| 28–30 | **-0.040 (0.004)***** | **-0.023 (0.004)***** | **-0.046 (0.005)***** | **-0.037 (0.004)***** | **-0.039 (0.006)***** | **-0.055 (0.006)***** |
| 30–32 | **-0.020 (0.004)***** | **-0.020 (0.004)***** | **-0.026 (0.004)***** | **-0.030 (0.003)***** | -0.018 (0.007) | **-0.053 (0.006)***** |
| 32–34 | **-0.037 (0.005)***** | **-0.029 (0.003)***** | **-0.043 (0.005)***** | **-0.038 (0.003)***** | -0.023 (0.008) | **-0.059 (0.006)***** |

**Supplementary Table S2.** Descriptive statistics for PDS items, HCP-D (ages 9–21). PDS items are scored 1–4 (height spurt, body hair, skin changes, voice deepening, facial hair, breast development) except menarche (pds_7, scored 1–4 in the HCP-D administration). The Tanner composite is a sex-specific combined score (range 5–20) derived from the applicable PDS items for each sex. r(age) is the Pearson correlation with age.

| **PDS variable** | **Applies to** | **N** | **Mean (SD)** | **Range** | **r (age)** |
| --- | --- | --- | --- | --- | --- |
| Height spurt | All | 595 | 2.84 (1.06) | 1–4 | 0.670 |
| Body hair growth | All | 595 | 2.83 (1.08) | 1–4 | 0.680 |
| Skin changes / acne | All | 595 | 2.44 (0.99) | 1–4 | 0.565 |
| Voice deepening | Males | 278 | 2.53 (1.02) | 1–4 | 0.739 |
| Facial hair growth | Males | 278 | 2.12 (1.04) | 1–4 | 0.782 |
| Breast development | Females | 317 | 2.83 (1.00) | 1–4 | 0.808 |
| Menarche | Females | 317 | 3.03 (1.40) | 1–4 | 0.742 |
| Tanner composite (males) | Males | 278 | 12.33 (4.21) | 5–20 | 0.818 |
| Tanner composite (females) | Females | 317 | 14.32 (4.77) | 5–20 | 0.842 |

**Supplementary Table S3.** Tanner stage as an independent predictor of RSI metrics, controlling for age, BMI, and sex. Models: RSI ~ age + BMI + sex + Tanner (combined, n=579); RSI ~ age + BMI + Tanner (sex-stratified, male n=271, female n=308). p-values shown are uncorrected. No regions are significant after Bonferroni correction across 12 tests (combined: 2 metrics × 6 regions) or 24 tests (sex-stratified: 2 metrics × 6 regions × 2 sexes).

| **Restricted Normalized Isotropic (RNI)** | | | | | | |
| --- | --- | --- | --- | --- | --- | --- |
| **Region** | **Combined β (SE) ×10⁻³** | **Combined p** | **Male β (SE) ×10⁻³** | **Male p** | **Female β (SE) ×10⁻³** | **Female p** |
| Amygdala | 0.41 (0.20) | 0.039 | 0.33 (0.30) | 0.271 | 0.65 (0.25) | 0.009 |
| Caudate | 0.18 (0.31) | 0.574 | 0.14 (0.44) | 0.752 | 0.38 (0.44) | 0.387 |
| Hippocampus | 0.41 (0.21) | 0.059 | 0.46 (0.32) | 0.161 | 0.52 (0.27) | 0.058 |
| Pallidum | 1.02 (0.46) | 0.029 | 0.18 (0.68) | 0.787 | 1.84 (0.63) | 0.004 |
| Putamen | 0.31 (0.23) | 0.171 | 0.11 (0.32) | 0.735 | 0.63 (0.30) | 0.038 |
| Thalamus | 0.30 (0.27) | 0.261 | 0.28 (0.40) | 0.474 | 0.49 (0.36) | 0.168 |
| **Hindered Normalized Isotropic (HNI)** | | | | | | |
| **Region** | **Combined β (SE) ×10⁻³** | **Combined p** | **Male β (SE) ×10⁻³** | **Male p** | **Female β (SE) ×10⁻³** | **Female p** |
| Amygdala | -0.28 (0.26) | 0.293 | -0.04 (0.39) | 0.914 | -0.65 (0.34) | 0.056 |
| Caudate | -0.39 (0.49) | 0.436 | -0.17 (0.70) | 0.809 | -0.66 (0.69) | 0.342 |
| Hippocampus | -0.98 (0.34) | 0.004 | -0.94 (0.50) | 0.059 | -1.23 (0.45) | 0.006 |
| Pallidum | -1.83 (0.94) | 0.052 | -0.75 (1.40) | 0.594 | -3.06 (1.27) | 0.016 |
| Putamen | -0.24 (0.25) | 0.340 | -0.25 (0.38) | 0.503 | -0.39 (0.33) | 0.242 |
| Thalamus | -0.85 (0.37) | 0.021 | -0.56 (0.56) | 0.318 | -1.35 (0.47) | 0.004 |

**Supplementary Table S4.** Variance in RSI metrics explained by age and pubertal stage. R² age only: RSI ~ age + BMI + sex. R² puberty only: RSI ~ BMI + sex + Tanner. R² age + puberty: RSI ~ age + BMI + sex + Tanner. ΔR² puberty | age: partial R² for Tanner added to the age model (F-test). ΔR² age | puberty: partial R² for age added to the puberty model (F-test). n=579 for all tests.

| **Restricted Normalized Isotropic (RNI)** | | | | | |
| --- | --- | --- | --- | --- | --- |
| **Region** | **R² age only** | **R² puberty only** | **R² age + puberty** | **ΔR² puberty \| age (p)** | **ΔR² age \| puberty (p)** |
| Amygdala | 68.3% | 57.7% | 68.6% | 0.74% (0.039) | 25.69% (6.34×10^-39^) |
| Caudate | 30.1% | 24.3% | 30.1% | 0.06% (0.574) | 7.67% (1.32×10^-11^) |
| Hippocampus | 56.3% | 50.3% | 56.6% | 0.62% (0.059) | 12.62% (1.40×10^-18^) |
| Pallidum | 63.5% | 52.0% | 63.8% | 0.83% (0.029) | 24.65% (3.46×10^-37^) |
| Putamen | 74.4% | 58.5% | 74.5% | 0.33% (0.171) | 38.56% (1.04×10^-62^) |
| Thalamus | 58.5% | 50.3% | 58.6% | 0.22% (0.261) | 16.70% (1.35×10^-24^) |
| **Hindered Normalized Isotropic (HNI)** | | | | | |
| **Region** | **R² age only** | **R² puberty only** | **R² age + puberty** | **ΔR² puberty \| age (p)** | **ΔR² age \| puberty (p)** |
| Amygdala | 35.4% | 33.3% | 35.5% | 0.19% (0.293) | 3.25% (1.34×10^-5^) |
| Caudate | 36.2% | 29.5% | 36.3% | 0.11% (0.436) | 9.61% (2.72×10^-14^) |
| Hippocampus | 30.0% | 29.9% | 31.0% | 1.40% (0.004) | 1.57% (0.003) |
| Pallidum | 57.8% | 47.3% | 58.1% | 0.66% (0.052) | 20.45% (2.20×10^-30^) |
| Putamen | 68.6% | 53.6% | 68.6% | 0.16% (0.340) | 32.32% (1.29×10^-50^) |
| Thalamus | 64.0% | 53.9% | 64.3% | 0.92% (0.021) | 22.68% (6.08×10^-34^) |

**Supplementary Table S5.** Statistical significance of individual PDS item effects on RSI metrics, controlling for age, BMI, and sex (age and BMI only for sex-specific items). Uncorrected p-values are shown. *p_Bonferroni_ < 0.05 with n_Tests_ = 84 (7 items x 2 metrics x 6 regions). M = Males only (n=271), F = Females only (n=308), all = Both sexes (n=579).

| **Restricted Normalized Isotropic (RNI)** | | | | | | |
| --- | --- | --- | --- | --- | --- | --- |
| **PDS item** | **Amygdala** | **Caudate** | **Hippocampus** | **Pallidum** | **Putamen** | **Thalamus** |
| Height spurt (all) | ↓ 0.639 | ↓ 0.393 | ↑ 0.962 | ↑ 0.678 | ↓ 0.786 | ↓ 0.414 |
| Body hair (all) | ↑ 0.101 | ↑ 0.885 | ↑ 0.322 | ↑ 0.276 | ↑ 0.651 | ↑ 0.110 |
| Skin / acne (all) | ↑ 0.151 | ↑ 0.312 | ↑ 0.052 | ↑ 0.065 | ↑ 0.128 | ↑ 0.777 |
| Voice deepening (M) | ↑ 0.112 | ↑ 0.115 | ↑ 0.074 | ↑ 0.136 | ↑ 0.194 | ↑ 0.350 |
| Facial hair (M) | ↑ 0.135 | ↑ 0.232 | ↑ 0.095 | ↑ 0.986 | ↑ 0.933 | ↑ 0.417 |
| Breast development (F) | ↑ 0.387 | ↑ 0.314 | ↑ 0.342 | ↑ 0.007 | ↑ 0.044 | ↑ 0.642 |
| Menarche (F) | ↑ 0.001 | ↓ 0.829 | ↑ 0.227 | ↑ 0.076 | ↑ 0.125 | ↑ 0.025 |
| **Hindered Normalized Isotropic (HNI)** | | | | | | |
| **PDS item** | **Amygdala** | **Caudate** | **Hippocampus** | **Pallidum** | **Putamen** | **Thalamus** |
| Height spurt (all) | ↓ 0.688 | ↓ 0.390 | ↓ 0.095 | ↓ 0.647 | ↓ 0.760 | ↓ 0.370 |
| Body hair (all) | ↓ 0.867 | ↓ 0.904 | ↓ 0.042 | ↓ 0.092 | ↑ 0.813 | ↓ 0.041 |
| Skin / acne (all) | ↓ 0.498 | ↓ 0.763 | ↓ 0.187 | ↓ 0.346 | ↓ 0.262 | ↓ 0.733 |
| Voice deepening (M) | ↓ 0.133 | ↓ 0.834 | ↓ 0.013 | ↓ 0.180 | ↓ 0.171 | ↓ 0.458 |
| Facial hair (M) | ↑ 0.244 | ↑ 0.639 | ↑ 0.826 | ↓ 0.801 | ↓ 0.737 | ↓ 0.409 |
| Breast development (F) | ↓ 0.324 | ↓ 0.697 | ↓ 0.258 | ↓ 0.095 | ↓ 0.700 | ↓ 0.107 |
| Menarche (F) | ↓ 0.014 | ↓ 0.135 | ↓ 0.001 | ↓ 0.053 | ↓ 0.181 | ↓ **1.73×10^-4^*** |

**Figure S1.** Variation of restricted normalized isotropic (RNI) signal fraction with age and sex (red=female, blue=male) before correction for an offset caused by the difference in protocols between HCP-D and HCP-YA. Curves show cubic and quadratic polynomial fits for HCP-D and HCP-YA protocols, respectively.

**Figure S2.** Variation of restricted normalized isotropic (RNI) signal fraction with age and sex (red=female, blue=male) after correction for an offset caused by the difference in protocols between HCP-D and HCP-YA. Curves show cubic and quadratic polynomial fits for HCP-D and HCP-YA protocols, respectively.

**Figure S3.** Variation of hindered normalized isotropic (HNI) signal fraction with age and sex (red=female, blue=male) before correction for an offset caused by the difference in protocols between HCP-D and HCP-YA. Curves show cubic and quadratic polynomial fits for HCP-D and HCP-YA protocols, respectively.

**Figure S4.** Variation of hindered normalized isotropic (HNI) signal fraction with age and sex (red=female, blue=male) after correction for an offset caused by the difference in protocols between HCP-D and HCP-YA. Curves show cubic and quadratic polynomial fits for HCP-D and HCP-YA protocols, respectively.

**Figure S5.** Variation of hindered normalized isotropic (HNI) component of diffusion with age and sex (red=female, blue=male) after protocol-offset correction.

**Figure S6.** Variation of Tanner composite score with age for males and females

**Figure S7.** Variation of individual Tanner subscores with age for males and females.
